# The long-term outcome of children with VP shunt and hydrocephalus: motor developmental outcome and QOL of patients with hydrocephalus is associated with the number of revisional procedures but is not impacted by the type of the valve

**DOI:** 10.3389/fsurg.2025.1530041

**Published:** 2025-01-27

**Authors:** Danielle S. Wendling-Keim, Hannah Luz, Elena Kren, Oliver Muensterer, Markus Lehner

**Affiliations:** ^1^ Department of Pediatric Surgery, Dr. von Hauner Children’s Hospital, Ludwig-Maximilians-University, Munich, Germany; ^2^ Department of Pediatric Surgery, Children’s Hospital Central Switzerland (KidZ), Luzerner Kantonsspital, University of Lucerne, Lucerne, Switzerland

**Keywords:** hydrocephalus, children, neurodevelopment, ventriculo-peritoneal shunt, revisional procedure, longterm outcome

## Abstract

**Purpose:**

Despite constant advances in ventriculo-peritoneal shunt systems, pediatric patients with hydrocephalus may present with neurodevelopmental delay. Therefore, we performed a study including a questionnaire, which aimed to analyze parameters that may have an impact on the cognitive function and quality of life of the pediatric patient with hydrocephalus.

**Methods:**

In this retrospective study, we included 81 patients aged 0–17 with hydrocephalus who were treated with a ventriculo-peritoneal shunt at a single institution. Demographic data, etiology of the hydrocephalus, type of valve implanted, any revision procedures and any complications were analyzed and the neurodevelopmental outcome, epilepsy and quality of life were assessed using a questionnaire sent to these patients. Statistical analysis was performed using SPSS. The significance level was set at *p* ≤ 0.05.

**Results:**

Questionnaires were sent to 81 patients who were treated at our institution over a mean retrospective study period of 18 years. Of these, 30 questionnaires were completed by the patients themselves or with the support of their families and included in the study. The etiology of the hydrocephalus as a non-controllable parameter did not affect the cognitive and motor development as well as the occurrence of epilepsy, cephalalgia and the quality of life. However, the number of revisions had a significant (*p* = 0.041) impact on the motor development of the child. The mean Wellbeing Five score was better with 19.63 in the group with no revisions whereas the score was 12.2 in the group with more than two revisional procedures. The type of the valve (adjustable or not adjustable) as a controllable parameter did not change any of the tested parameters (*p* > 0.05).

**Conclusion:**

The number of revisional procedures significantly affected the motor development of pediatric patients with a ventriculo-peritoneal (VP) shunt calling for further research to improve VP shunt systems as well as surgical procedures like endoscopic third ventriculo-cisternostomy (ETV) in the future.

## 1 Introduction

The standard treatment for hydrocephalus is the implantation of a ventriculo-peritoneal shunt (VP shunt)^1^. Despite of ongoing improvements of VP shunts the prognosis of the pediatric patients regarding their quality of life and neurodevelopment remains unclear to date. There is literature pointing to a decreased quality of life in patients with VP shunts (1). However, previous studies have reported the lack of data regarding the quality of life and long-term outcome of patients with hydrocephalus and VP shunts (2). Additionally, a metanalysis in 2021 suggests an impaired neurodevelopment in children with ventriculo-peritoneal shunts (3). Other studies also suggest a compromised progress concerning various aspects of brain development including the visuomotor integration and coordination while more studies focusing on advanced technologies including neuroendoscopic lavage are needed to evaluate their role for an improved prognosis of these patients (4–6). To date it is not clear which parameters influence the quality of life and the motor and cognitive development of patients with hydrocephalus and VP shunts.

Therefore, it is the goal of this study to identify factors including the etiology of the hydrocephalus, the type of the valve and the number of revisions, that may change the cognitive and motor development as well as the quality of life in patients with VP-shunts.

## 2 Patients and methods

We conducted a retrospective cohort study including a questionnaire which addressed neurodevelopment, quality of life and satisfaction with the VP shunt system. From a pool of 117 patients who presented with hydrocephalus to our tertiary care hospital at the age of 0–18 years, 36 patients were excluded from the study due to bilateral VP shunts and missing data regarding the primary surgery at another institution or drop out during the follow up period. 81 patients who were retrospectively analyzed over a mean time period of 18 years received a questionnaire by mail and of these, 30 forms were completed and sent back to us by the patients and their families. At the time of the completion of the questionnaire, the patients were between 9 and 36 years old. Consent for the questionnaire was given by the parents and, if applicable, by the patients themselves. The questionnaire included the topics neurodevelopment, cephalalgia, epilepsy, and quality of life including the “Wellbeing Five” questionnaire by the WHO (WHO-5). Most questions were multiple choice questions. The complete questionnaire can be found in the Supplementary (S1).

All data were irreversibly anonymized. Data were expressed as means ± standard deviation and subjected to Student's unpaired *t*-test and Spearman's rank correlation. A level of *p* < 0.05 was considered significant.

This study was performed in line with the principles of the Declaration of Helsinki. Approval was granted by the Ethics Committee.

## 3 Results

### 3.1 Patient demographics

In this study, 81 patients with hydrocephalus and VP shunts were contacted by mail and received a questionnaire. Of these, 30 patients (37%), 15 male and 15 female patients, returned their completed questionnaires and were included. The hydrocephalus was caused by intracerebral hemorrhage (ICH) in 50.0% of cases (*n* = 14), associated with meningomyelocele (MMC) in 13.3% of cases (*n* = 4) and congenital in 20% (*n* = 6) of cases. In 10% (*n* = 3) of the patients, the hydrocephalus occurred after an infection, in one child (3.3%) after traumatic brain injury (TBI) and in 6.7% (*n* = 2) the etiology was unknown. An outline of the baseline characteristics of the patients included in this study is shown in Table 1.

**Table 1:** Baseline characteristics of the patients enrolled in this study.

Baseline characteristics		*N*
Age at questionnaire assessment	9–36 years	
Sex	Female	15
	Male	15
Preterm/fullterm		13/17
Type of valve	Adjustable	7
	Non-adjustable	23
School enrolment	Standard school	13
	Special school	16
	Not enrolled	1
Cognitive development	Regular	11
	Delayed	6
	Learning difficulties	5
	Cognitive impairment	8
Motor development	Regular	16
	Assistive devices	4
	Wheel chair	10
Epilepsy	No epilpsy	15
	No seizures under medication	12
	Refractory epilepsy	3
Etiology	Intracerebral hemorrhage	14
	Congenital	6
	MMC	4
	Infectious	2
	Idiopathic	2
	Posttraumatic	1
Number of revisions	No revisions	8
	One revision	3
	Two revisions	11
	More than two revisions	5

### 3.2 Overall neurodevelopmental outcome

Of the patients who completed the questionnaire, 43.4% (*n* = 13) attended regular schools. 53.3% (*n* = 16) joined special schools whereas one patient (3.3%) was not able to enroll in any kind of school. The overall neurodevelopmental course was evaluated and rated as age-appropriate development in 36.7% (*n* = 11) children. 16.7% of the patients (*n* = 5) presented with learning disabilities and 46.7% (*n* = 14) showed a delayed neurodevelopment or cognitive impairment. The motor development was normal in 53.3% of the patients from the questionnaire (*n* = 16). Further, 33.3% (*n* = 10) children had a wheelchair and 13.3% (*n* = 4) were dependent on other assistive devices. 12 (40%) of the patients were seizure free under medication whereas three (10%) children suffered from seizures despite of their medication, and the other 50% presented without any kind of epilepsy (Table 1).

### 3.3 The etiology of the hydrocephalus does not affect the neurodevelopmental outcome of the child but changes the duration of intermittent cephalalgias

**Figure 1:**
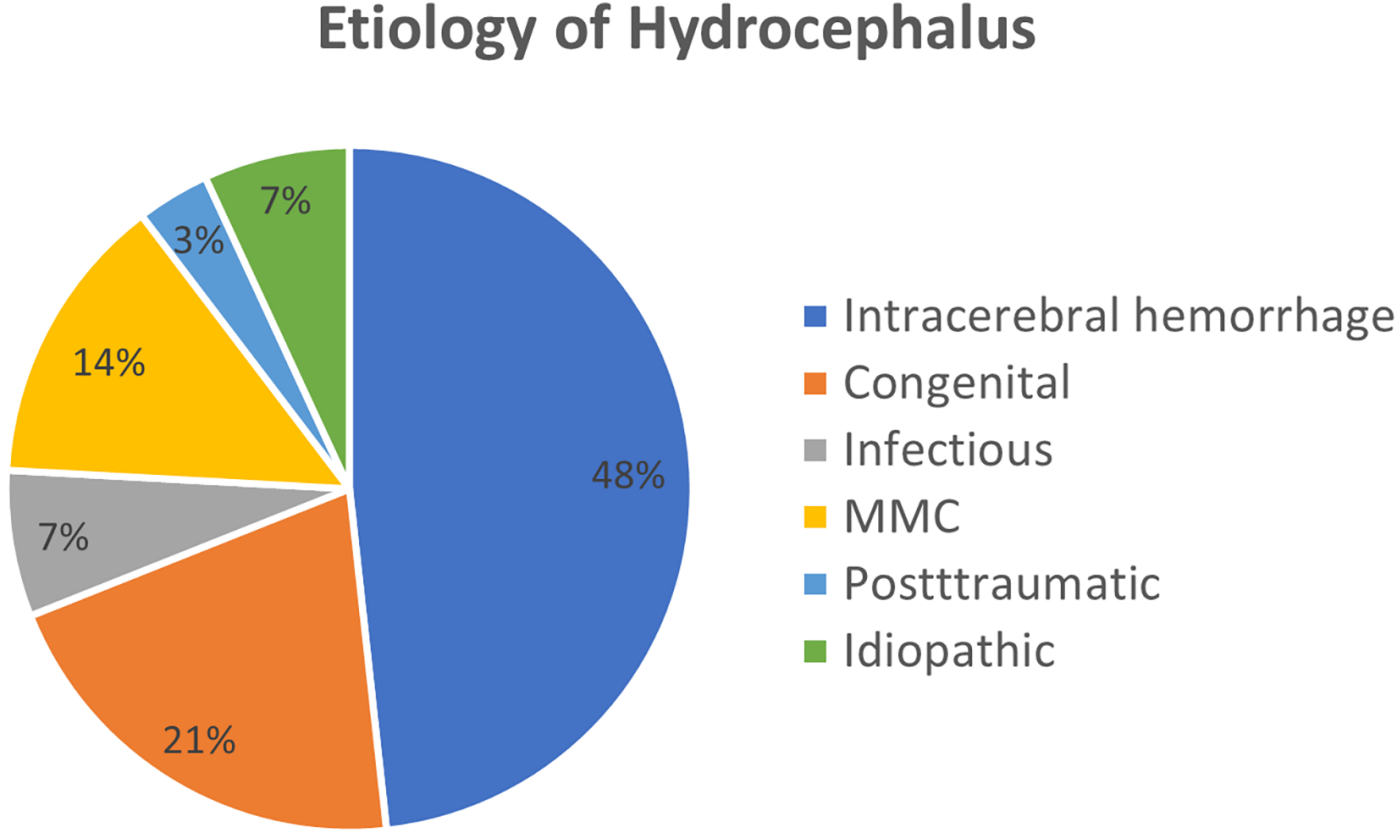
Etiology of the hydrocephalus. Here we show the etiology of the hydrocephalus of the patients in this study.

**Figure 2:**
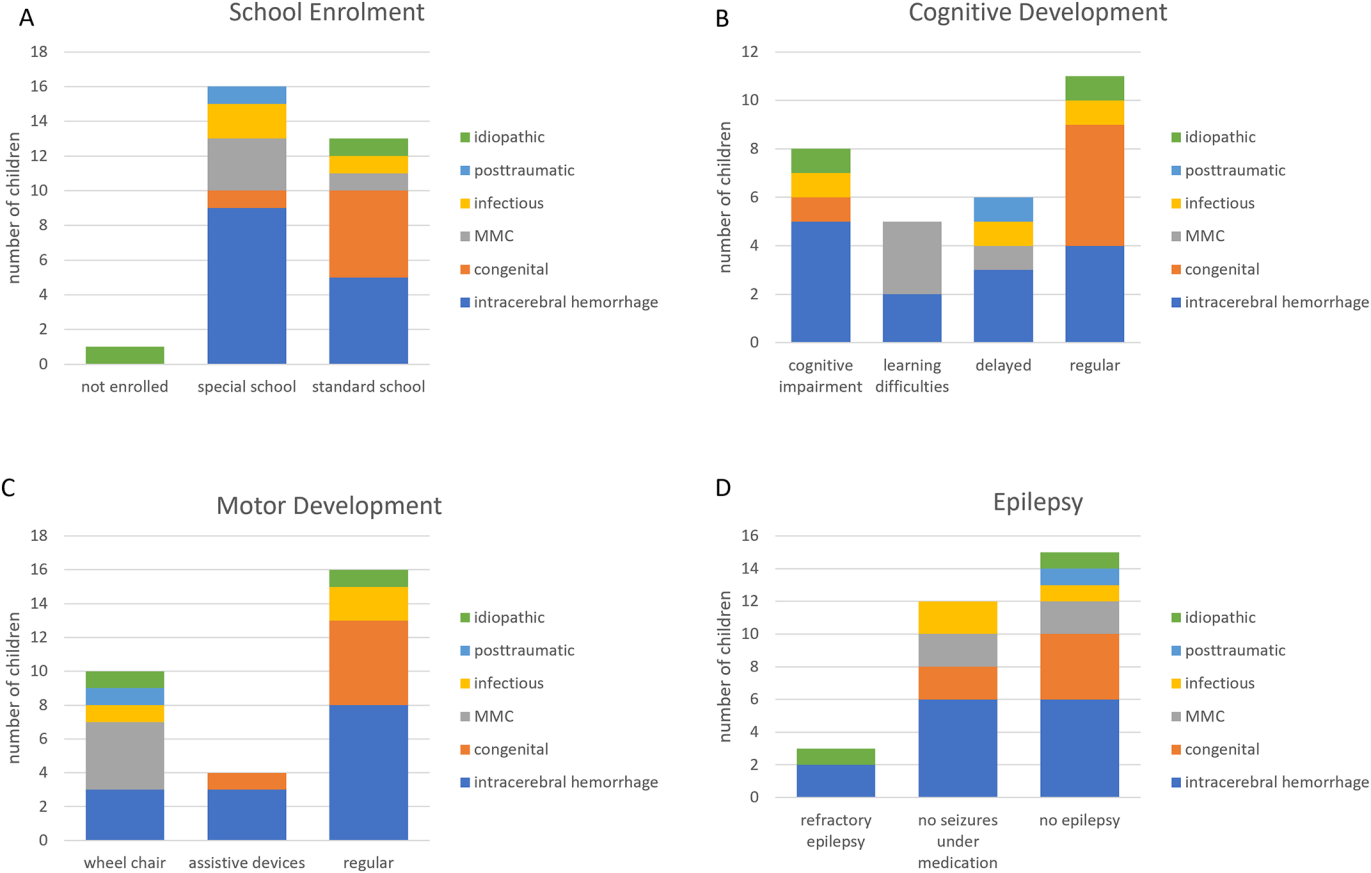
Impact of the etiology of the hydrocephalus on the neurodevelopment. The etiology did not have any impact on the school enrollment **(A)**, cognitive **(B)** and motor development **(C,D)** or epilepsy of the patients.

An overview of the cause of hydrocephalus of the children in this study is demonstrated in Figure 1. Of the patients who responded to the questionnaire, 47% presented with post-hemorrhagic hydrocephalus (*n* = 14). Of these, nine children attended special schools, and five patients attended regular schools. Further, four children showed an age-appropriate neurodevelopment, whereas eight patients were neurodevelopmentally delayed or deficient and two presented with learning disabilities. Also, all four patients with MMC required special schools and 75% of them presented with learning disabilities or neurological deficits. In contrast, patients with congenital hydrocephalus mostly (83.3%) attended regular schools and showed no neurodevelopmental delay or deficit, only one of these patients needed a special school and was not age-appropriately developed. Within the remaining groups of patients with infectious (*n* = 3) or posttraumatic hydrocephalus (*n* = 1) or hydrocephalus with unknown cause (*n* = 2) two patients with age-appropriate development attended regular schools; the other children went to special schools and presented with an impaired neurodevelopment. However, ANOVA analysis revealed that the etiology of the hydrocephalus did not have a significant (*p* = 0.29) impact on the schools the patients were able to attend (Figure 2A). Accordingly, we also did not find any significant difference (*p* = 0.073) of the cognitive development due to different etiologies of the hydrocephalus (Figure 2B). The motor development of the patients and the development of epilepsy was not altered by the etiology of the hydrocephalus (*p* = 0.121/*p* = 0.64, respectively; Figures 2C,D). To highlight the largest group of patients with intracerebral hemorrhage we color-coded the etiology in Figure 2 showing these patients in blue.

Further, the questionnaire revealed that the frequency and intensity of cephalalgias in patients with VP Shunts was not influenced by the etiology of the hydrocephalus (*p* = 0.631; *p* = 0.193, respectively). Only the duration of cephalalgias was affected by the cause of the hydrocephalus, with traumatic brain injury leading to significantly longer lasting symptoms (*p* = 0.009).

### 3.4 The number of VP-shunt revisions affects the motor development

**Figure 3:**
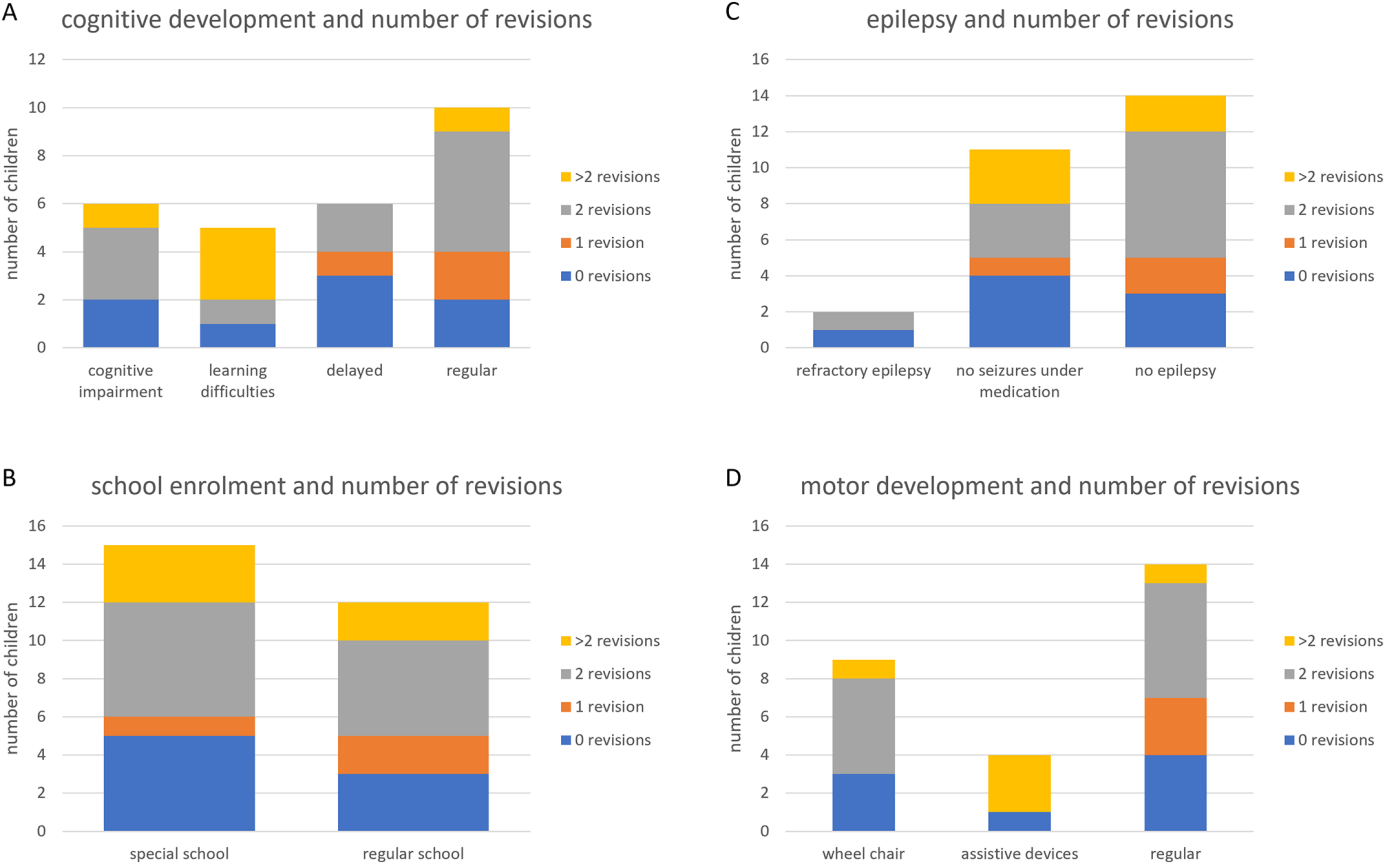
The role of the number of revisional surgeries on the development of the patients. Repeated revisions of the VP shunt system impaired the motor development of the children **(D)**, but not their cognitive development, school enrollment or course of epilepsy **(A–C)**.

The patients from the questionnaire were analyzed regarding the impact of the number of revisions on their neurodevelopment. Three patients were excluded from the analysis due to missing data. 26.7% of patients did not need any revision, 10% of patients had one revision during the follow up period, 36.7% of the children needed two revision surgeries and 16,7% children had a change of the complete shunt system or parts of it more than 2 times (Table 1). The cognitive development of the children and the type of schools they attended as well as the occurrence and severity of their epilepsy were not impacted by the number of revisions (*p* = 0.31; *p* = 0.59, 0.816, Figures 3A–C, respectively). Further, neither the frequency, nor the duration or the intensity of cephalalgias was changed by the number of revisions of the VP Shunts (*p* = 0.662; *p* = 0.685; *p* = 0.533, respectively). However, motor development significantly (*p* = 0.041) worsened with increasing numbers of revisions leading to the need for a wheelchair and assistive devices in these patients. Of the children with more than two revisions, only one had a regular motor development (Figure 3D).

### 3.5 The type of the valve does not affect the neurodevelopment or the occurrence of cephalalgias

To investigate the impact of a controllable parameter we analyzed the data to determine whether an adjustable valve could provide an advantage for the neurodevelopment of the patients in comparison to fixed-pressure valves. We assessed the types of school attended, the cognitive and motor development as well as the occurrence of epilepsy to evaluate the development of the child. Here we found no significant difference (*p* = 0.386; *p* = 0.145; *p* = 0.339; *p* = 0.359, respectively) between patients with an adjustable valve or a fixed valve. Further, frequency (*p* = 0.511), duration (*p* = 0.524) and intensity (*p* = 0.650) of cephalalgias were unchanged by the type of the valve when we compared adjustable and non-adjustable valves.

### 3.6 The number of revisions may impair the quality of life in children with VP shunts

**Figure 4:**
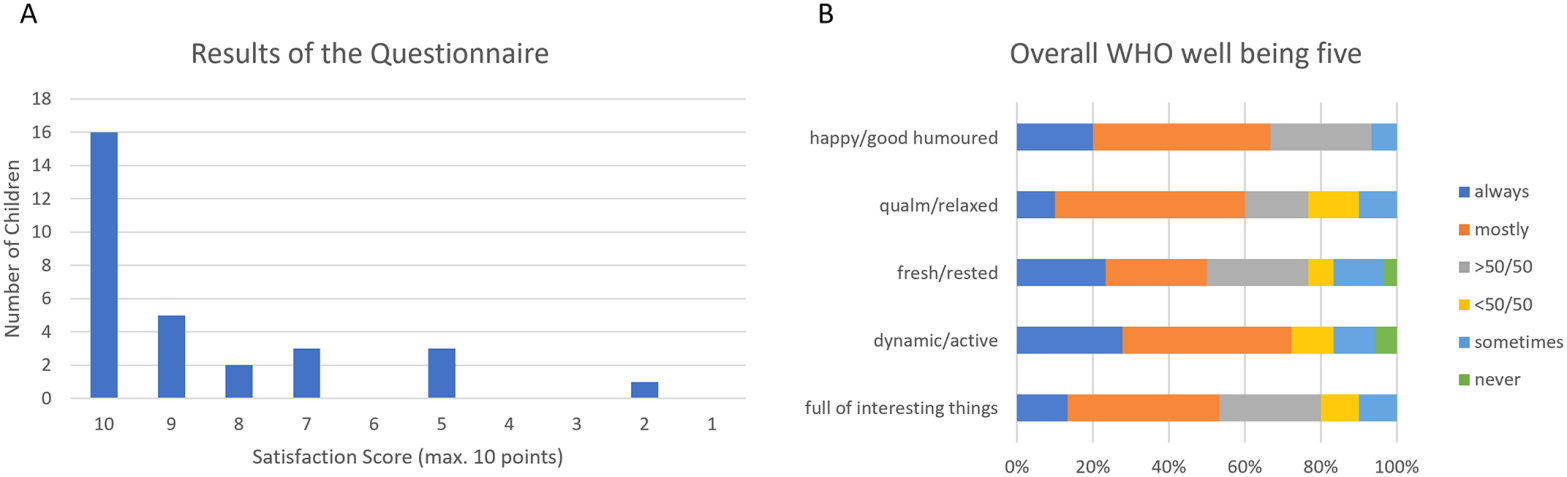
Overall results of the questionnaire. The overall satisfaction score is shows high values in most patients **(A)**. The Overall WHO Wellbeing Five confirms the good results of the satisfaction score **(B)**.

The questionnaire included the assessment of the perception of the patients regarding their impairment in daily-life-activities and their satisfaction with their VP shunt. More than half of the patients (53.3%) who returned the questionnaire were fully satisfied (10/10) with their VP shunt system and the mean grade of satisfaction was reported with 8.6/10 points. However, nine patients rated the system with 8 points or less out of 10, and the level of satisfaction of the patients who did not return the questionnaire remains unknown (Figure 4A). In detail, seven (23.3%) patients complained about impairments of their daily life due to the VP system while 10 patients reported deficiencies during sports and travel.

The quality of life was further assessed by implementing the WHO Wellbeing Five in our questionnaire. The lowest score achieved by one of the responding patients was 8 out of 25 according to a score of 32/100 Wellbeing Five score while the average score was 17.1/25 according to a Wellbeing Five score of 68.4/100 (Figure 4B).

**Figure 5:**
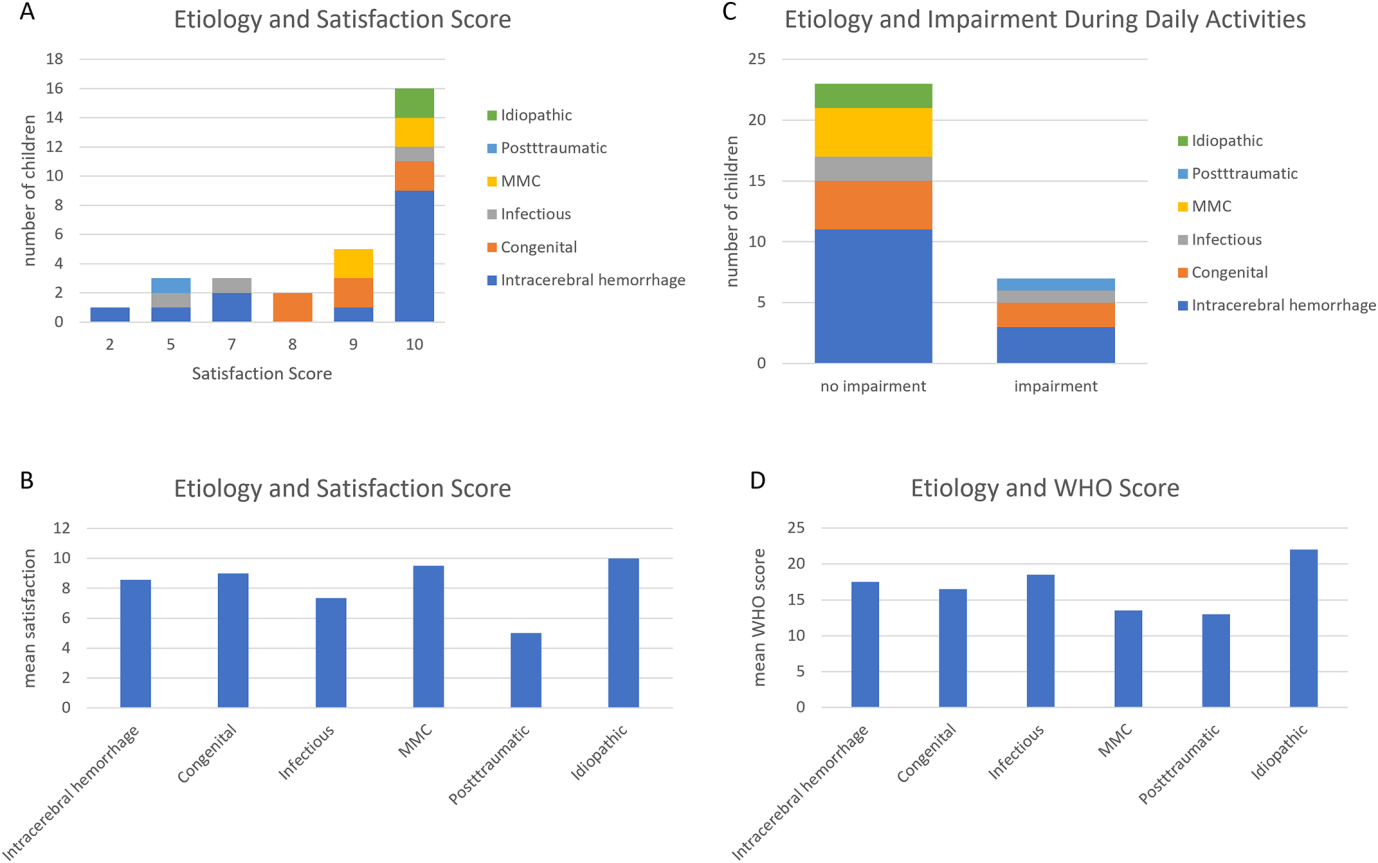
Etiology of the hydrocephalus and Qol. The etiology of the hydrocephalus does not change the satisfaction score **(A,B)**, impairment during daily activities **(C)** and WHO **(D)**.

In a next step, we assessed the association of the patient satisfaction and impairment during daily activities or travels with controllable and non-controllable parameters. We found that the etiology of the hydrocephalus as a non-controllable factor did not change the patients' satisfaction, impairment during their daily activities or travels and sports (*p* = 0.18; 0.34; 0.68, respectively). Neither did the etiology affect the results of the Wellbeing Five questionnaire (*p* = 0.344) (Figures 5A–D).

**Figure 6:**
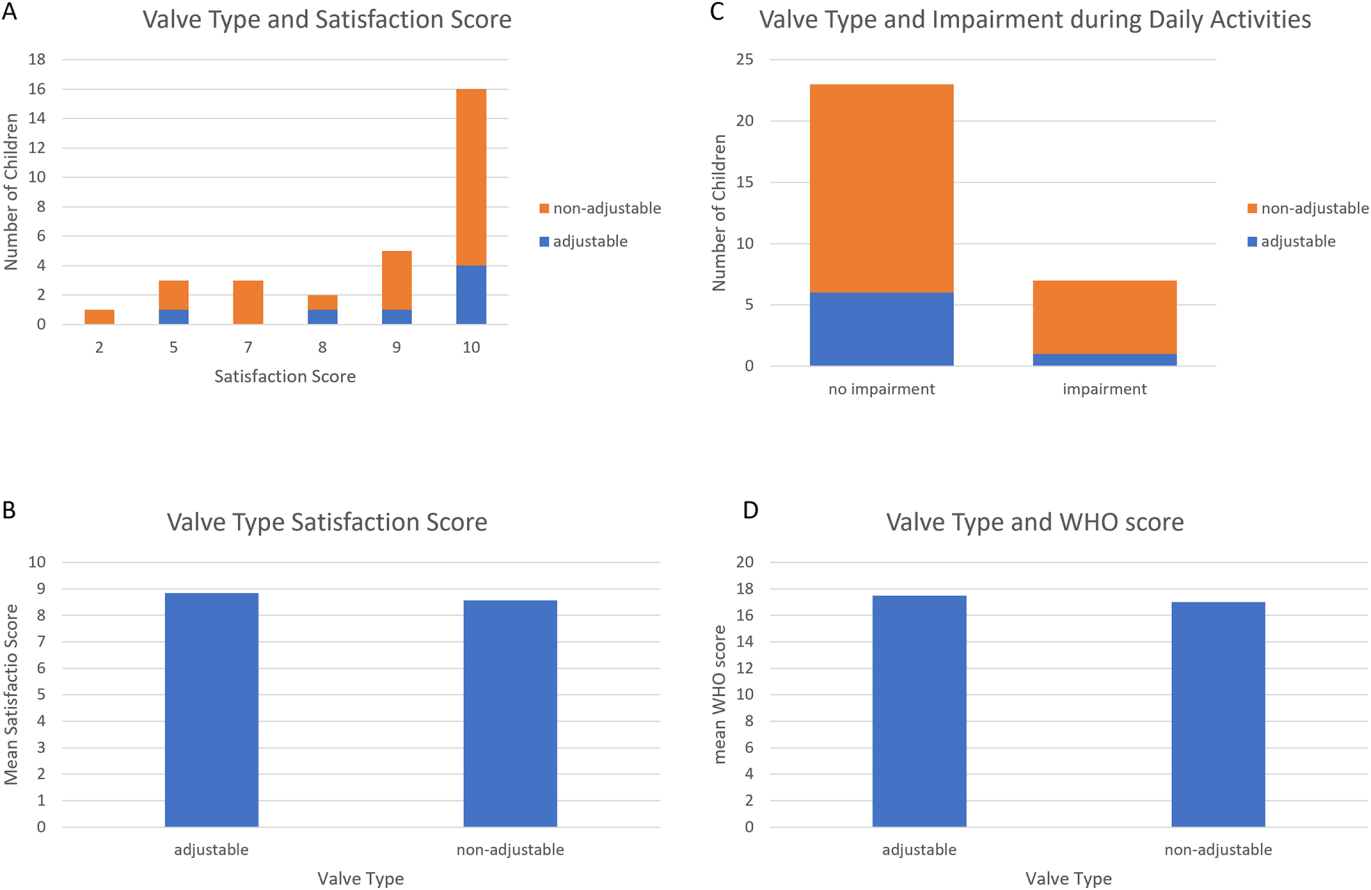
Valve type and Qol. The type of the valve did not change the Qol assessed in the questionnaire **(A–D)**.

Further, the type of the valve (adjustable or fixed) of the VP Shunt as a controllable factor did not influence patient satisfaction, wellbeing according to the WHO Wellbeing Five or feeling impaired in any activities (*p* > 0.05; Figure 6).

**Figure 7:**
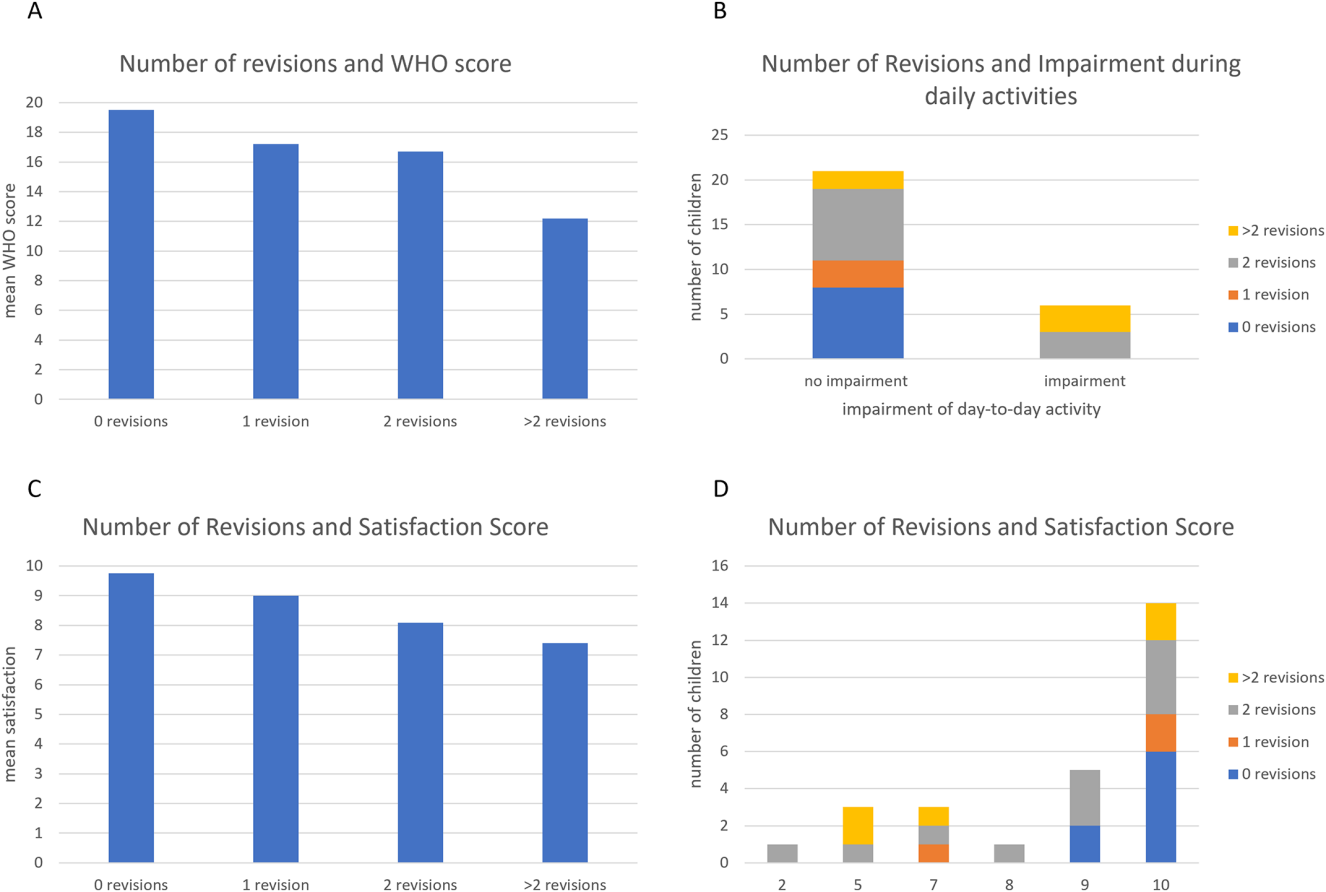
Number of revisions and Qol. An increased number of revisions did not change the satisfaction scores **(A,C,D)**, but tended to impair daily activities **(B)**.

However, the number of revisional surgical procedures showed a trend towards a significant difference: The mean Wellbeing Five score was 19.63 in the group with no revisions whereas the score was 12.2 in the group with more than two revisional procedures (Figure 7A). Patients with two or more revisional procedures tended to have lower (*p* = 0.06; Figure 7A) mean Wellbeing Five Scores than the patients with less than two revisions and also tended to feel more impaired in their daily activities (*p* = 0.059, Figure 7B). Nevertheless, no difference was seen regarding the patient satisfaction score (*p* = 0.51; Figures 7C,D) and travel activities (*p* = 0.145) according to the questionnaire.

## 4 Discussion

In this study, we analyzed 30 questionnaires that were returned from 15 male and 15 female patients with hydrocephalus. Due to follow up visits at our outpatient clinic, we were able to have our patients under surveillance for a period of between 9 years and 36 years and to reveal the long-term outcomes after VP shunt implantation. The main etiology of the hydrocephalus of the patients studied was an intracerebral hemorrhage in preterm infants. Interestingly, the underlying cause of the hydrocephalus did not change the neurodevelopmental outcome or the occurrence of epilepsy or cephalalgias for patients in this study. Even if different courses of neurodevelopment may be expected in children with different etiologies of the hydrocephalus (7, 8), our analysis showed that MMC or traumatic brain injury as the etiology did not have any impact on the outcome specifically in patients of this study, so we were able to compare these patients and analyze for other parameters. Although there may be a bias regarding the return of the questionnaire in a way that the parents of the healthier patients may have returned the questionnaire at a higher rate than patients who are more impaired by the VP shunt, we can see from the baseline table that the majority of the patients in this study attend special schools and therefore do have special needs and impairments. In detail, 43.3% of the patients went to regular schools, 53.3% attended special schools and 3.3% were not enrolled at a school. Nevertheless, we found that the duration of cephalalgias was the only parameter that was influenced by the etiology of the hydrocephalus with traumatic brain injury being followed by longer episodes of cephalalgias than the other causes. Almost half of the patients analyzed (14/30) suffered from posthemorrhagic hydrocephalus. This is especially interesting in the light of two studies that show that posthemorrhagic hydrocephalus in premature infants is heterogeneous (7) and a relevant co-morbidity with the overall outcome being poor, especially in children with leukomalacia after IVH (8).

Further, a recently published study by Sobana et al. (3) demonstrated that children with non-infectious hydrocephalus and a CSF diversion (VP-shunt) are at a significant risk for disabilities and mental as well as motoric developmental delay. Nevertheless, until now we cannot safely differentiate to which extent the problems with the VP shunt are responsible for the impairment they suffer from in comparison to the underlying disease. Another major factor under discussion is the number of revisions possibly influencing the patients' development and wellbeing. Although our data showed that the cognitive development as well as the occurrence of cephalalgias and epilepsy did not correlate with the number of revisions as opposed to previous studies (9–11) we detected increasing deficiencies of the motor development in children who had more than one revision procedure. This is especially relevant in the light of another study by Kulkarni et al. (12) and by our group (13) showing that the rate of revision procedures needed in VP-Shunt patients remains high, therefore calling for alternative methods.

From the data that were available from this study we did not find any difference between patients with adjustable and non-adjustable valves. However, the group with adjustable valves was a lot smaller (*n* = 7) than the group with non-adjustable valves (*n* = 23). From the numbers that we collected we saw that 71.4% of patients with an adjustable valve attended a regular school and showed normal motor development as opposed to 34.8% and 47.8%, respectively, of the patients with non-adjustable valves although the results were not statistically significant due to the small number of patients. This observed tendency of benefits from adjustable valves has been described previously in a study by Bock et al. (14), but only for infants during very early development, so that further studies are needed to clarify potential advantages of adjustable valves in the future. Strikingly, the majority of patients (53.5%; *n* = 16) were fully satisfied (10 points) with their shunt implanted and the overall satisfaction was rated at a mean of 8.6/10. The high satisfaction score demonstrates that the patients were hardly affected by the mechanical device. This is a major point and should be presented to the patient and parents prior to shunt placement. Nevertheless, an impairment by the shunt system was sensed in special situations like travel or sports in 33.3% of the patients in this study whereas even higher rates of feeling impaired was noted in another previously published article by Beez et al. (15). This discrepancy between the high satisfaction with the VP shunt system and the felt relatively high restriction during sports and travel may reflect that the described high contentment may be confined to daily activities. Further studies focusing on the complexity of patient satisfaction are needed in the future, especially in the light of the role of physical activity for patients with disabilities (16).

The quality of life according to the Wellbeing Five questionnaire by the WHO of the patients of our study population was satisfying with an average score of 68.4/100. The only parameter influencing the patients' quality of life was the number of revisions. More than two revisions led to a near significant impact on the quality of life with impaired daily activities thus now difference was seen in patient satisfaction or traveling activities: motor development significantly (*p* = 0.041) changed with the number of revisions with more revisions leading to the need for wheelchairs and assistive devices in these patients. This is especially relevant in the context of depression amongst children and teenagers. A large study by Allgaier et al. (17) has found that 3.6% of children aged 9–12 years and 11.7% of teenagers from 13 to 16 years suffered from depression based on the Wellbeing Five questionnaire. Among the hydrocephalus patients from our study, 16.7% of patients scored under the threshold of 13 points pointing to a higher risk for depression in these children. Especially patients without a revision had a Wellbeing Five score of 19.6 whereas patients with more than two revisions presented with a score of only 12.2. This suggests that the VP shunt may become a relevant burden for patients in case of repeated revision procedures despite of the generally good results from the questionnaire. Therefore, the surgical management may need improvement. Endoscopic Third-Ventriculostomy (ETV) may enhance treatment by drainage without placement of a mechanical device or by flushing the ventricles after hemorrhage before implantation of a reservoir or shunt. In some recent studies (18–20) the proportion of patients needing valve-depending treatments is decreasing, so that even in children with post hemorrhagic hydrocephalus an increasing percentage of children may not need a mechanical device. In the light of previous studies that found that 84.5% of patients with VP shunts needed at least one revision (21) the treatment of hydrocephalus possibly needs further modification. Our findings that point to an impaired development and quality of life in children who need more than one revision may lead to altered clinical decisions, especially in resource-limited settings, with possibly alternative treatment options including endoscopic ventriculostomy.

### 4.1 Limitations of the study

The assessment of satisfaction and quality of life in general and especially in under school aged children is challenging. Subjectivity as well as the ability to understand the questions and expressing the answers may lead to recall or assessment bias. Further, the socioeconomic status of the families and their ability to initiate early intervention, care and support may influence the development of the children and was not captured in this survey. Nevertheless, the importance of this factor is known at our institute and the socioeconomic situation is routinely assessed and addressed during follow-up visits at our institution. However, comorbidities apart from the etiology of the hydrocephalus need to be addressed in a further study. Also, the questionnaire assessed the neurodevelopment and QoL of the patients at a specific point in time which may change during further follow ups after the end of this study. Although we were able to follow a relatively large group of patients over a very long follow up period of 9–18 years, the number of patients was low and statistical analysis limited. Further, the study was retrospective, and adjustable valves were added only during the course of time. Therefore, future prospective studies with a larger number of participants but with a comparable follow up time are needed. In addition, selection bias may occur since we do not have any knowledge of the reasons why not all questionnaires were returned. The response rate of the questionnaire was 37%. One reason for the low return may be that no incentives or benefits were offered. Another reason may have been that the questionnaires were sent by mail to the families of the patients while some of these patients have already been transitioned to an adult clinic making the feedback to the childen's hospital less attractive and relevant for their current treatment. To analyze the reason for non-participation, a telephone interview may have been more appropriate. Further, in future studies, the evaluation of the quality of life may be examined repeatedly at different ages in order to analyze the impact of age on the answers.

However, according to data previously published the response rate of surveys varies between 10.3% and 61% (13). Further, even with a small sample size (i.e., less than 500) 20%–25% response rates are sufficient for a valid analysis according to a meta-analysis by Wu et al. (22).

## 5 Conclusion

The frequency of revisional procedures significantly impacted the motor development of pediatric patients with VP shunt in this study. Additionally, we observed a trend towards a reduced quality of life in patients undergoing repeated shunt revisions. These findings call for improvements of the surgical and multiprofessional management of these patients.

## Data Availability

The raw data supporting the conclusions of this article will be made available by the authors, without undue reservation.
