# Supplementary material for: The long-term outcome of children with VP shunt and hydrocephalus: motor developmental outcome and QOL of patients with hydrocephalus is associated with the number of revisional procedures but is not impacted by the type of the valve
Source: Front Surg. 2025 Jan 27;12:1530041. doi: 10.3389/fsurg.2025.1530041 (PMC11808032; doi:10.3389/fsurg.2025.1530041)
Supplement: Supplementary file 1 [file Table1.docx]

Child’s name: ……………………………………………… Date of Birth: …………………………………
Birth weight: ……………………………………………… Gestational age: …………………

Siblings: ☐yes ☐no Twins: ☐yes ☐1^st^ twin ☐2^nd^ twin ☐no

Date: (1^st^ Implantation of shunt valve) …………………………………… Revisions: ☐yes ☐no

Current shunt valve type: ………………………………………….…..

#### Development

Schooling: ☐ regular school ☐ special needs school ☐ School for the disabled ☐ no schooling

Misceallaneous: …………………………………………………

Mental abilities: ☐ age appropriate ☐delayed ☐learning difficulties ☐ cognitive impairment

Misceallaneous: …………………………………………………

Motor development: ☐ age appropriate ☐ ambulatory with assistive devices ☐ wheelchair required

Misceallaneous: ……………………………………….…….…..

What sports does your child play?: …………………………………………………

#### Epilepsy

Does your child suffer from any form of cerebral seizures (epilepsy)? ☐yes ☐no

Is your child seizure-free with medication? ☐Yes, since ………………………… ☐no

How frequently have seizures occured in the last month/year? …………… per months or per year

Did the seizures occur in certain situations? If yes, when?

#### Headaches

On average, how often has your child experienced headaches in the last 6 months?

- daily ☐ at least every week ☐ at least twice a month ☐infrequent

How long do headaches usually last?

Up to 1 hour ☐2-4 hours ☐ up to one day ☐2 days ☐longer than 2 days
How severe is the pain on average?

☐10 ☐9 ☐8 ☐7 ☐6 ☐5 ☐4 ☐3 ☐2 ☐1 ☐0

Worst pain imaginable no pain

If your child has a headache …? never seldom sometimes frequent often

is he/she bothered by loud noise

is he/she bothered by bright light

is he/she neauseous

is he/she vomiting

is the pain worsening if he/she moves around

is he/she experiencing flickering in front of the eyes

is he/she having a tense neck/throat

is he/she dizzy

Misceallaneous …………………………………

The headaches happen after…

| Weather/climate change | |  | |
| --- | --- | --- | --- |
| Loud noise | |  | |
| Watching a lot of TV/playing on the computer | |  | |
| Not drinking enough | |  | |
| On hot days | |  | |
| Strong physical activity | |  | |
| Not sleeping enough | |  | |
| On weekends / at the start of the holidays | |  | |
| Fights / stress (in school, social environment) | |  | |
| Misceallaneous ……………………………………. |  | |  |

Coping strategies

Take a short break      Lie down (in a dark room)

Medication      Misceallaneous …………………………………….

What medication is your child taking for the headache?

| Which one, in what dosage? | very seldom | sometimes | often | every day |
| --- | --- | --- | --- | --- |
| ……………………………………………………………….. | ☐ | ☐ | ☐ | ☐ |
| ……………………………………………………………….. | ☐ | ☐ | ☐ | ☐ |
| ………………………………………………………………. | ☐ | ☐ | ☐ | ☐ |
| ……………………………………………………………….. | ☐ | ☐ | ☐ | ☐ |

#### Life with the shunt

Overall, how satisfied are you with the shunt?

☐10 ☐9 ☐8 ☐7 ☐6 ☐5 ☐4 ☐3=>☐2 ☐1 ☐0

Very satisfied very unsatisfied

Does the shunt affect your everyday life? If yes, how?

Are you taking precautions before traveling, doings sports? If yes, which ones?

On how many days per month does your child experience symptoms that you attribute to the shunt? What complaints/symptoms are these?

#### WHO-Quality of life questionnaire („Wellbeing Five“)

| **During the last two weeks…** | **The whole time** | **Mostly** | **More than half the time** | **Less than half the time** | **Sometimes** | **Never** |
| --- | --- | --- | --- | --- | --- | --- |
| **... your child was happy and in a good mood** | ☐5 | ☐4 | ☐3 | ☐2 | ☐1 | ☐0 |
| **… your child felt calm and relaxed** | ☐5 | ☐4 | ☐3 | ☐2 | ☐1 | ☐0 |
| **… your child felt well rested when he/she woke up** | ☐5 | ☐4 | ☐3 | ☐2 | ☐1 | ☐0 |
| **… your child felt dynamic and active** | ☐5 | ☐4 | ☐3 | ☐2 | ☐1 | ☐0 |
| **… everydaylife was full of things that interest your child** | ☐5 | ☐4 | ☐3 | ☐2 | ☐1 | ☐0 |
